# Source identification and potential health risks from elevated groundwater nitrate contamination in Sundarbans coastal aquifers, India

**DOI:** 10.1038/s41598-024-54646-0

**Published:** 2024-02-20

**Authors:** Subodh Chandra Pal, Tanmoy Biswas, Asit Kumar Jaydhar, Dipankar Ruidas, Asish Saha, Indrajit Chowdhuri, Sudipto Mandal, Aznarul Islam, Abu Reza Md. Towfiqul Islam, Chaitanya B. Pande, Edris Alam, Md Kamrul Islam

**Affiliations:** 1 Department of Geography, The University of Burdwan, Purba Bardhaman, West Bengal 713104 India; 2 Ecology and Environmental Modelling Laboratory, Department of Environmental Science, The University of Burdwan, Purba Bardhaman, West Bengal 713104 India; 3 Department of Geography, Aliah University, 17 Gorachand Road, Kolkata, West Bengal 700 014 India; 4 Department of Disaster Management, Begum Rokeya University, Rangpur, 5400 Bangladesh; 5 Department of Development Studies, Daffodil International University, Dhaka, 1216 Bangladesh; 6 Indian Institute of Tropical Meteorology, Pune, India; 7 New Era and Development in Civil Engineering Research Group, Scientific Research Center, Al-Ayen University, Thi-Qar, Nasiriyah, 64001 Iraq; 8 Faculty of Resilience, Rabdan Academy, 22401 Abu Dhabi, United Arab Emirates; 9 Department of Geography and Environmental Studies, University of Chittagong, Chittagong, 4331 Bangladesh; 10 Department of Civil and Environmental Engineering College of Engineering, King Faisal University, 31982 AlAhsa, Saudi Arabia

**Keywords:** Nitrate contamination, Groundwater pollution, Probable health risk, Sundarbans, Environmental sciences, Hydrology

## Abstract

In recent years groundwater contamination through nitrate contamination has increased rapidly in the managementof water research. In our study, fourteen nitrate conditioning factors were used, and multi-collinearity analysis is done. Among all variables, pH is crucial and ranked one, with a value of 0.77, which controls the nitrate concentration in the coastal aquifer in South 24 Parganas. The second important factor is Cl^−^, the value of which is 0.71. Other factors like—As, F^−^, EC and Mg^2+^ ranked third, fourth and fifth position, and their value are 0.69, 0.69, 0.67 and 0.55, respectively. Due to contaminated water, people of this district are suffering from several diseases like kidney damage (around 60%), liver (about 40%), low pressure due to salinity, fever, and headache. The applied method is for other regions to determine the nitrate concentration predictions and for the justifiable alterationof some management strategies.

## Introduction

Groundwater is an essential resource for all living beings in the world and plays a key role in reducing the water crisis and increasing agricultural productivity and industrial activity^1^. Different research articles showed that about 780 million people face water scarcity globally^2^. In various activities like agriculture, industry, drinking water and other domestic purposes, groundwater plays a key role in fulfilling such criteria^1^. Yet, coastal aquifers are at risk at various activities like the intrusion of salt water,over-exploitation of groundwater, contamination of nitrate and different trace metals due to agricultural activities^3^. Cattle barns and sewage effluent are the other major source of nitrate concentration in coastal areas (Bernhard et al.^4^). In the coastal area of West Bengal, the major source of nitrate contamination is the decomposition of organic matter in the soil, agricultural fertilizer used in the field, and industrial effluents. In this coastal area, groundwater is the only potable source^3^. The matter of nitrate concentration and related issues is recently a great threat all over the world such as in Asia^5–7^, America^8^, Australia^9^. In densely polluted coastal areas countries like India, groundwater quality is contaminated due to nitrate concentration (Pal et al.^28^). In agricultural fields, excess use of livestock residue is another vital cause of nitrate concentration in the lower or shallow aquifer^9,10^. In a coastal area, the groundwater level is shallower and the only drinking water source so nitrate contamination increases daily^11,12^. The most common contaminant in groundwater has been nitrates since 1970; nitrate is a natural component found in groundwater, but contamination occurs when it exceeds 3 mg/l. The United States Environmental Protection Agency (USEPA) sets the level of nitrate in groundwater for blue baby syndrome at > 10 mg/l. It ensures that below 10 mg/l is considered safe for everyone for drinking purposes^13^. An excess nitrate increase in groundwater (more than 50 mg/l) causes diseases like blue baby syndrome (WHO^14^). Human life quality can be improved by improving drinking water quality^15^; recently, industrialization and fertilizers used in agricultural fields have increased the nitrate concentration and related health issues in different parts of India as well as different countries in the world (Asadi et al.^16^). In the Indian agricultural field for farm production, huge amount of nitrogen is used as a result, nitrogen increases in groundwater, river water, pond etc. (Pal et al.^28^; Rehman et al.^17^). In groundwater, concentration of nitrate rapidly increases due do its more solubility and mobility. Various side effects are reported due to its ill effects like, blue baby syndrome (infants), gland problems and colon cancer^18^. Different factors and processes are responsible for assessing nitrate concentration in groundwater (Pal et al.^28^).

Different researchers used different strategies for determining the pollution status in various parts of the world, like the index method and interpolation method^19^. According to Narany et al.^20^, a sampling point which is very depth is needed for the interpolation method. Expert knowledge is needed to make an accurate position in a statistical model^21^. According to Islam et al.^3^, Groundwater nitrate concentration data is very rare till now and collection of data from the field is very cost-effective so it is critical to evaluate nitrate concentration and its effects on different aspects. To eradicate this problem, we used different modelling approaches and techniques and noticed the measures of groundwater nitrate concentration. In our study, we used the Mean Decrease Accuracy Method (MDA), the Logistic Regression model (LR) in the RS-GIS environment, different software including ArcGIS 10.8, IBM SPSS 20, and various statistical methods. Fourteen causative factors have been considered for determining the concentration of nitrate, which is pH, chloride, arsenic, fluoride, electrical conductivity, magnesium, nitrate, potassium, temperature, sulphate, phosphate, sodium, salinity, depth, and bicarbonate.

According to Pitchaikani et al.^22^, the coastal aquifer in West Bengal is facing nitrate problems, which leads to heath-related issues in the populations of that region. In this coastal region, few researchers gave attention to determining the nitrate concentration and its effect on human health. This research determines the NO_3_− concentration through the LR method and health-related issues. Therefore, the main objective of this work is to determine the hydro-chemical properties of groundwater and probable health risks in coastal aquifers of the Sundarbans region. In our research, we prepared a NO_3_− susceptibility map to show the health-related issues and determine the reasons for elevated nitrate concentration and health hazards. The novelty and objectives of this research work are that people can easily find the pattern of nitrate concentration in this study area and find the nitrate hazard distribution map, which some other researchers have not created till now. With the help of this research work, the government can take required action and strategy for reducing the ill-effects of nitrate concentration.

## Study area

**Figure 1:**
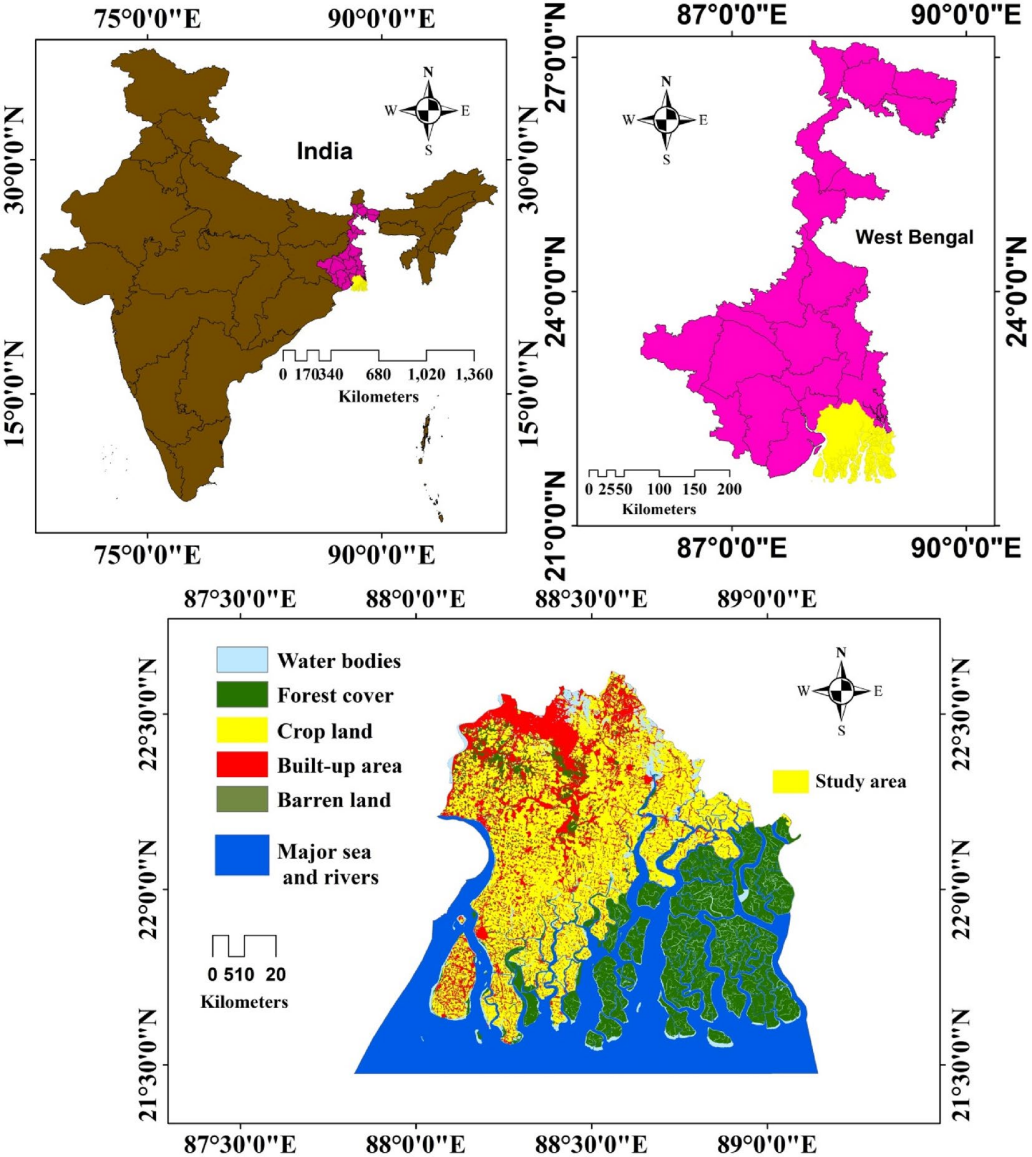
Location map of the study area (this map was generated using ArcGIS, version: 10.3.1, www.esri.com/arcgis).

There are two coastal districts in West Bengal: East Medinipur and South 24 Parganas. Study revealed, South 24 Parganas is the largest district in of area (9960 sq. km) and second largest as per population concern. On 1st March 1986, this district was separated into two parts, North and South 24 Parganas. The locational extent of this district is 22° 12′ 13″ and 22° 46′ 55″ North latitude and 87° 58′ 45″ and 88° 22′ 10″ East latitude which is shown in (Fig. 1). Kolkata district is in the north, and Howrah and East Medinipur are in the West. It has 2042 villages. This district shares a long international border (Bangladesh) to the east and southern parts of the Bay of Bengal. The largest mangrove ecosystem is in the south, south–west, south–east and eastern parts of this district. This district has five subdivisions, including Kakdwip, Baruipur, Alipore Sadar, Diamond Harbour and Canning with seven municipalities and 29 community blocks and 111 census towns (Census 2011). The total population of this district is 8,161,961, almost equal to the country as Honduras or the Virginia state of USA. The population density of inhabitants is 819 per sq. km. The population growth rate over 2001–2011 is 18.2%. According to the census 2011, the sex ratio is 956/1000 males, and a 77.51% is literacy rate. Baruipur northern Plain and Kulpi-Diamond Harbour Plain is found in the north part of this district ie, almost 5–6 m above sea level.

In this portion, the land creation process is also going on. Hot and humid climatic condition is found all over the district throughout the year, and rainfall occurs by the southwest monsoon wind. The highest temperature occurred in Diamond Harbour (37 °C) and the lowest temperature occurred at 9 °C (Census 2011). This region is famous for its natural environment, like the Sundarbans, which is well known as the habitat of the Royal Bengal Tiger. Sundarbans is the world's largest mangrove forest area. It is revealed that this district is a deposit of various natural resources like groundwater, oil, and natural gas conducting different tests. Due to the presence of other rivers and bills, khals and the Bay of Bengal; this region’s soil is divided into two types: saline and non-saline. Deposit soil of Ganga is saline free, which is rich in nutrients which is favourable for the cultivation of different crops like rice, wheat, barley, maize, etc.

### Hydrogeological setting

Total South 24 Parganas district is situated under the Gangetic delta; the southern portion of this district a large are is covered by the Sundarban Biosphere Reserve (SBR), and rivers flowing over this area like Matla, Thakuran, Raidighi, Bidya, Raimangal and Saptamukhi etc. Islands are situated in this district i.e., Sagar Island, Fraserganj, Lothian Island, Bulcherry, Halliday Island, Dalhousie Island, and Bangaduni Island at the mouth of the river Gosaba of these few Islands are submerged under seawater. The study area also includes the primary intertidal deltaic mass and the coast sand associated with estuaries and tidal streams; alluvial and marine silt of the Quaternary era make up the majority of the South 24 Parganas district's geological features in the Bengal basin^23^. Das et al.^24^ state, that although delta formation is still ongoing, the northern portion of the South 24 Parganas is a component of the active delta zone; the restricted aquifer serves as the primary supply of drinking water in this area, and deeper aquifers have also been observed there. According to Datta and Kaul^25^, depending on their vertical position, aquifers can range in depth from 160 to 335 m, which are notable sources of drinking and irrigation water; tertiary silt and alluvium from the Pleistocene to the present comprise the majority of the aquifer strata in this region. This region significantly falls under the lower ganga basin area of the Holocene Sediments predominantly collected in lacustrine, marine, and fluvial settings^26^. The porous alluvial and coastal sediments in the area allowed undesirable pollutants to seep and infiltrate into the groundwater aquifer^27^; at a depth of 160 to 400 m below the surface, the aquifer is composed primarily of freshwater layers, whereas the shallow aquifer, about 60 m below the surface, is dominated by salty water. In the study region, parent rock played a noteworthy role in salinity intrusion, hydro-geological interaction and cation exchange which significantly impact water quality^28^.

### Methodology and data sources

**Figure 2:**
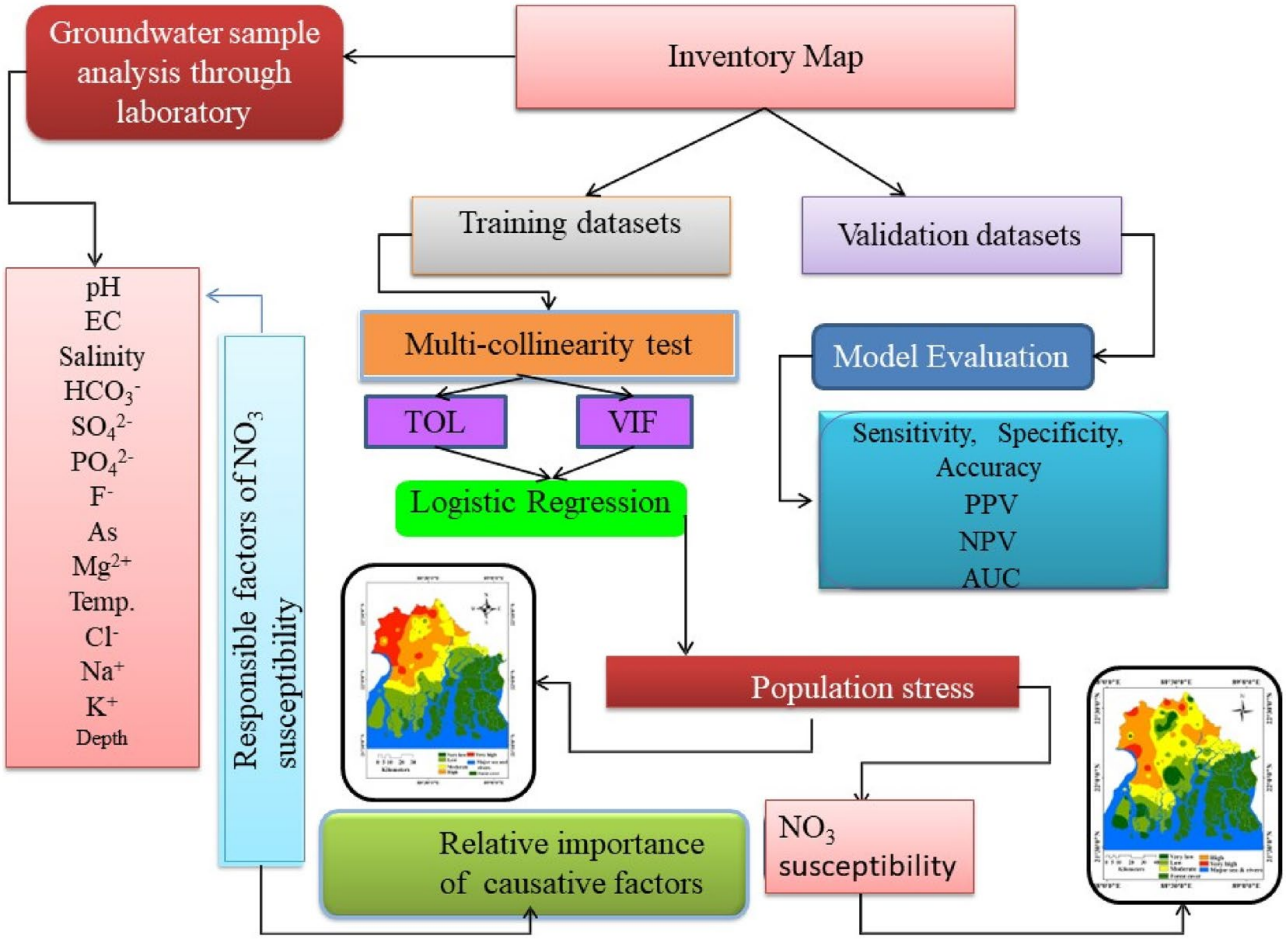
Methodological flow chart.

Big data is required for conducting the research, a total of 58 samples have been collected throughout this district. By using Google Earth-pro software, we determined the tube well samples in this region. GPS was used for documentation and recording the data. Before gathering the water, disconnecting the standing water 10 to 15 min, groundwater was pumped. -density washed bottles were used to collect water (Jaydhar et al.^26^);After that, the samples were immediately transferred to the Burdwan University laboratory and stored at below 5 °C for laboratory analysis of the hydro-chemical properties of groundwater. Cations and anions were determined by ion chromatography using Dionex ICS-90. The inductive coupled plasma mass spectrometry method is used for analysing As (Islam et al.^3^. Quality control tools and critical procedures of the lab were used for quality assurance of groundwater. To conduct this research, Logistic Regression (LR) method is used and ArcGIS 10.2.4 is used for the thematic layer of different parameters like depth of water, the temperature of water, salinity, EC, pH, K^+^, Mg^2+^, Na^+^, As, F^−^, Cl^−^, HCO_3_−, PO_4_^2−^, SO_4_^2−^, and NO_3_−. The susceptibility map of NO_3_− and human health hazard map was prepared by ArcGIS 10.2.4 software. Piper diagram and USSL diagram is crafted for describe the water quality. The flow chart of the methodology is shown in Fig. 2.

## Logistic regression

One important and commonly used modelss is Logistic Regression (LR); in several applications, various researchers cite the LR model on their research topic (Pradhan and Lee^29^). In real situations, it is challenging to use; the severe assumption was defined by the LR model, which is measured the difficulty of the approaches in this study. Several statistical approaches based on the LR model can overwhelm this difficulty and formulate a straightforward approach which uses different analyses like bivariate such as frequency ratio^30^. Still LR method is much suitable than other methods, several drawbacks are present in this method. To solve this problem, multiple studies apply bivariate analysis of LR; despite some drawbacks, one advantage of the LR model is that it can calculate the discrete and continuous data separately or together. LR model was done by using the “Statistical Package for Social Science (SPSS) V 15 programme”. By using the following equation, we calculate LR

1 $${\text{P}}=\frac{{\text{exp}}(z)}{1+{\text{exp}}(z)}$$

where, P represents the subsequent equation can calculate particular observational possibility possibilities and z –

2 $${\text{z}}=\mathrm{\beta o}+\upbeta 1{\text{X}}1+\upbeta 2{\text{X}}2+\dots \mathrm{\beta nXn}$$

where $\mathrm{\beta o}$ represents algorithm intercept, n and X1 represent conditioning factors, $\upbeta 1$ represents independent variable contribution.

### Health risk estimation (HRE)

The health risk of the people was estimated by adopting the subsequent equations introduced by (US EPA^31^):

3 $$CDIi=\frac{Cw*IR*EF*ED}{BW*AT}$$

where ‘CDIi’ represents ingestion of chronic regular dose of specific trace element (μg/kg/day); ‘Cw’ implies concentration of heavy metal in potable water (μg/l); ‘IR’ suggests the consumption rate of drinking water (0.70 for children and 21.00 for adults); ‘EF’ reveals rate of exposure; ‘ED’ denotes duration of exposure (6 years for children and 30 years for adults); ‘BW’ suggests the body weight of person (15 kg for children and 70 kg for adults) whereas ‘AT’ represents the average time of exposure (2,190 days for children and 10,950 days for adults).

Absorption of CDI dermal calculated through the following expression (Eq. 4) (US EPA^31^):

4 $${\text{CDId}}=\frac{{\text{CW}}*{\text{SA}}*{\text{Kp}}*{\text{ET}}*{\text{EF}}*{\text{ED}}*{\text{CF}}}{Bw*AT}$$

where, ‘CDId’ indicates dermal of day-to-day dosage of chronic trace elements (μg/kg/day); ‘SA’ signifies exposure of skin area; ‘Kp’ represents permeability coefficient; ‘ET’ suggests time of contaminants exposure rate (h/day) and ‘CF’ means factors responsible for units of conversion (L/cm^3^).

Hazard quotient (HQ) of every trace element was measured by applying the successive equation (Eq. 5):

5 $${\text{HQ}}=\frac{CDI}{RfD}$$

RfD of every contaminant was obtained from regulations of (US EPA^31^).

Probable health risk of the people was estimated through the subsequent equation (Eq. 6):

6 $${\text{HI}}={\sum }_{i=1}^{n}HQ$$

where, HI is Health Risk Index.

## Results

### Statistical analysis of causative factors

#### Physical properties of groundwater in coastal aquifers

**Figure 3:**
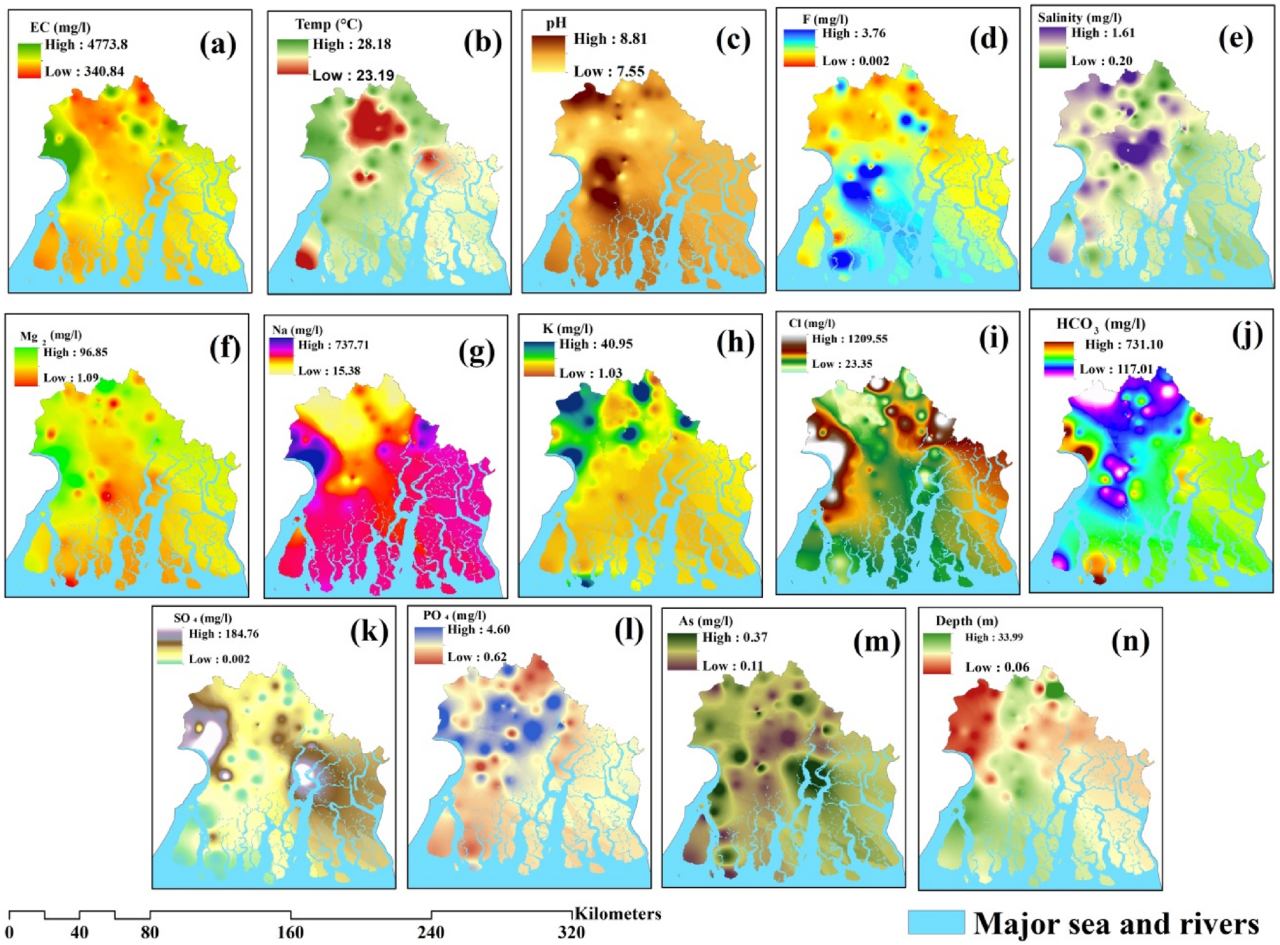
Causative factors for nitrate susceptibility; (**a**) EC, (**b**) Temperature, (**c**) pH, (**d**) F^−^, (**e**) Salinity, (**f**) Mg^2+^, (**g**) Na^+^, (**h**) K^+^, (**i**) Cl^−^, (**j**) HCO_3_−, (**k**) SO_4_^2−^, (**l**) PO_4_^2−^, (**m**) As, (**n**) Depth (all this map was generated using ArcGIS, version: 10.3.1, www.esri.com/arcgis).

Each of the conditioning elements that have been chosen has unique physical and chemical characteristics that play a significant role in regulating the water quality of a given location. This is especially true in the complex coastal zone, where the quality of aquifers is equally influenced by both land and seawater. Generally speaking, the distributional pattern of several the conditioning factors chosen for this study varies during the investigation rather than remaining constant. The descriptive statistics state the distributional pattern of all adopted conditioning factors mentioned in Table 1. The conditioning factors, including EC, temperature, and pH varies 340.84–4773.8 (Fig. 3a), 23.19 °C–28 °C (Fig. 3b), and 7.55–8.81(Fig. 3c); accordingly, the highest concentration of EC was observed in Diamond Harbour I and II block along with this north–western and north–eastern part were experienced with higher temperature; salinity and groundwater depth ranges from 0.20–1.61 mg/l (Fig. 3e) to 0.06–33.39 m (Fig. 3n). Another critical component like F^−^, average value is 0.79 and ranges from 3.76 to 0.002 mg/l (Fig. 3d), primarily found in southern part of Namkhana and Kulpi region; average values of Mg^2+^, Na^+^ and K^+^ are 36.34 mg/l, 182.38 mg/l and 8.276 mg/l (Table 1) which ranges from 96.85 to 1.09 mg/l (Fig. 3f), 737.71 to 15.38 mg/l (Fig. 3g) and 40.95 to 1.03 mg/l (Fig. 3h) respectively. As, PO_4_^2−^, and SO_4_^2−^ are very distinctive hydro-chemical properties of groundwater, average values are 0.204 mg/l, 2.29 mg/l and 31.87 mg/l (Table 1); values range from 0.37 to 0.11 mg/l (Fig. 3m), 4.60 to 0.62 mg/l (Fig. 3l) and 184.76 to 0.002 mg/l (Fig. 3k) accordingly. In addition to this, Fig. 3i and j represent spatial distribution of CI and HCO_3._ The distributional pattern is very uneven throughout the entire study region; the highest proportion of salinity was observed in the middle part and northern part of this study area, whereas the concentration of Mg^2+^ is high in the western part of this district, which also another important causative factor; Na^+^ is high near Diamond Harbour II, and K+ mostly found in north and north and north–eastern part of this study region.

**Table 1:** Descriptive statistics of selected parameters.

	Range	Min	Max	Mean		Std. Deviation	Variance	Skewness		Kurtosis	
	Statistic	Stat	Statistic	Statistic	Std. Error	Statistic	Statistic	Statistic	Std. Error	Statistic	Std. Error
NO_3_− (mg/l)	41.0	0.0	41.0	6.276	1.1544	8.7915	77.291	2.857	.314	8.032	.618
As	.259	.118	.377	.20438	.007774	.059205	.004	.733	.314	− .055	.618
PO_4_^2−^ (mg/l)	3.980	0.623	4.613	2.294	0.138	1.054	1.111	.721	.314	− .481	.618
SO_4_^2−^ (mg/l)	184.0	1.0	185.0	31.879	5.0489	38.4512	1478.494	1.991	.314	4.554	.618
HCO_3_−	616.0	116.0	732.0	373.690	15.8276	120.5393	14529.727	.366	.314	.415	.618
Cl^−^ (mg/l)	1191.0	21.0	1212.0	198.190	24.8682	189.3907	35868.823	3.145	.314	13.800	.618
K^+^	40.0	1.0	41.0	8.276	1.0735	8.1753	66.835	2.849	.314	8.175	.618
Na^+^	724.0	15.0	739.0	182.586	15.0624	114.7118	13158.808	2.025	.314	8.547	.618
Mg^2+^	96.0	1.0	97.0	36.345	2.4215	18.4415	340.090	1.248	.314	2.886	.618
F^−^	3.79	.01	3.80	.7922	.10259	.78133	.610	2.130	.314	4.484	.618
pH	1.27	7.55	8.82	8.1929	.03821	.29098	.085	.229	.314	− .131	.618
EC (μS/cm)	4444.0	338.0	4782.0	1202.517	90.8917	692.2109	479155.973	2.871	.314	11.889	.618
Depth (m)	34.06	.05	34.11	7.7967	.70241	5.34938	28.616	1.861	.314	9.137	.618
Temp. (°C)	5.01	23.19	28.20	26.4584	.19565	1.49000	2.220	− .822	.314	− .859	.618
Salinity	1.450	.206	1.656	.41119	.027258	.207591	.043	3.998	.314	22.480	.618
Valid N (listwise)

#### Correlation among hydro-chemical parameters

**Figure 4:**
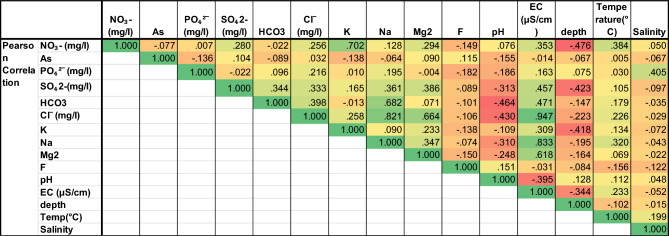
Correlation among all variables.

All groundwater samples were characterised with distinctive hydro-chemical compositions. Using Pearson's correlation matrix analysis in SPSS software, these physicochemical characteristics were mentioned in Fig. 4. The validity of the results is demonstrated by the statistical analysis, which also included descriptive statistics and Pearson's correlation, which logically supported the decision to use of parameters. After analysing all groundwater samples, several conditioning factors are considered, including As, PO_4_^2−^, SO_4_^2−^, HCO_3_−, Cl^−^, K^+^, Na^+^, Mg^2+^, F^−^, pH, EC, depth, temperature, and salinity. Our research shows that some causative factors have a highly positive and negative correlation to each other. Figure 4 states NO_3_− and K^+^ have significant interdependence (0.702) to each other; Cl^−^ strongly correlated with Na^+^ (0.821), EC (0.947) and Mg^2+^ (0.664), whereas Na^+^ have distinctive interdependence with HCO_3_− (0.982) and EC (0.833). Apart from these, all parameters have interdependence with each other but are very negligible. This result helps us to understand the interdependence among all adopted conditioning factors; it works very beneficial in determining the appropriate causative factors in current research work.

### Multi-collinearity assessment of variables

We used multi co linear analysiWe used multi co linear analysis to study the linear relationship among variables to check the linear relationship among variables. We used fourteen hydro-chemical properties for analysis. The variance Inflation Factor (VIF) and Tolerance of the sample are shown in the Table 2. VIF and tolerance are highly negatively correlated with each other. If the VIF value increases, then the Tolerance value also decreases. In case of EC, Cl^−^ and As, the Tolerance values are 0.056, 0.041 and 0.021, which is below the threshold value. In the case of Na^+^ the highest VIF value is 8.75. In our study, the VIF value extends within 10, so we can say that there is no multi-collinearity problem among all variables.

**Table 2:** Multi–collinearity values for several explanatory factors.

Factors	Collinearity analysis	
	TOL	VIF
K^+^	0.636	1.573
PO_4_^2−^ (mg/l)	0.491	2.038
As (μg/l)	0.021	1.226
SO_4_^2−^ (mg/l)	0.605	1.652
HCO_3_− (mg/l)	0.286	3.498
Cl^−^ (mg/l)	0.041	6.375
Na^+^ (mg/l)	0.101	8.75
EC (μS/cm)	0.056	7.92
pH	0.445	2.247
F^−^ (mg/l)	0.778	1.28
Temperature (°C)	0.738	1.355
Water depth (m)	0.607	1.64
Mg^2+^ (mg/l)	0.336	2.98
Salinity	0.721	1.38

### Population pressure related stress on water quality

**Figure 5:**
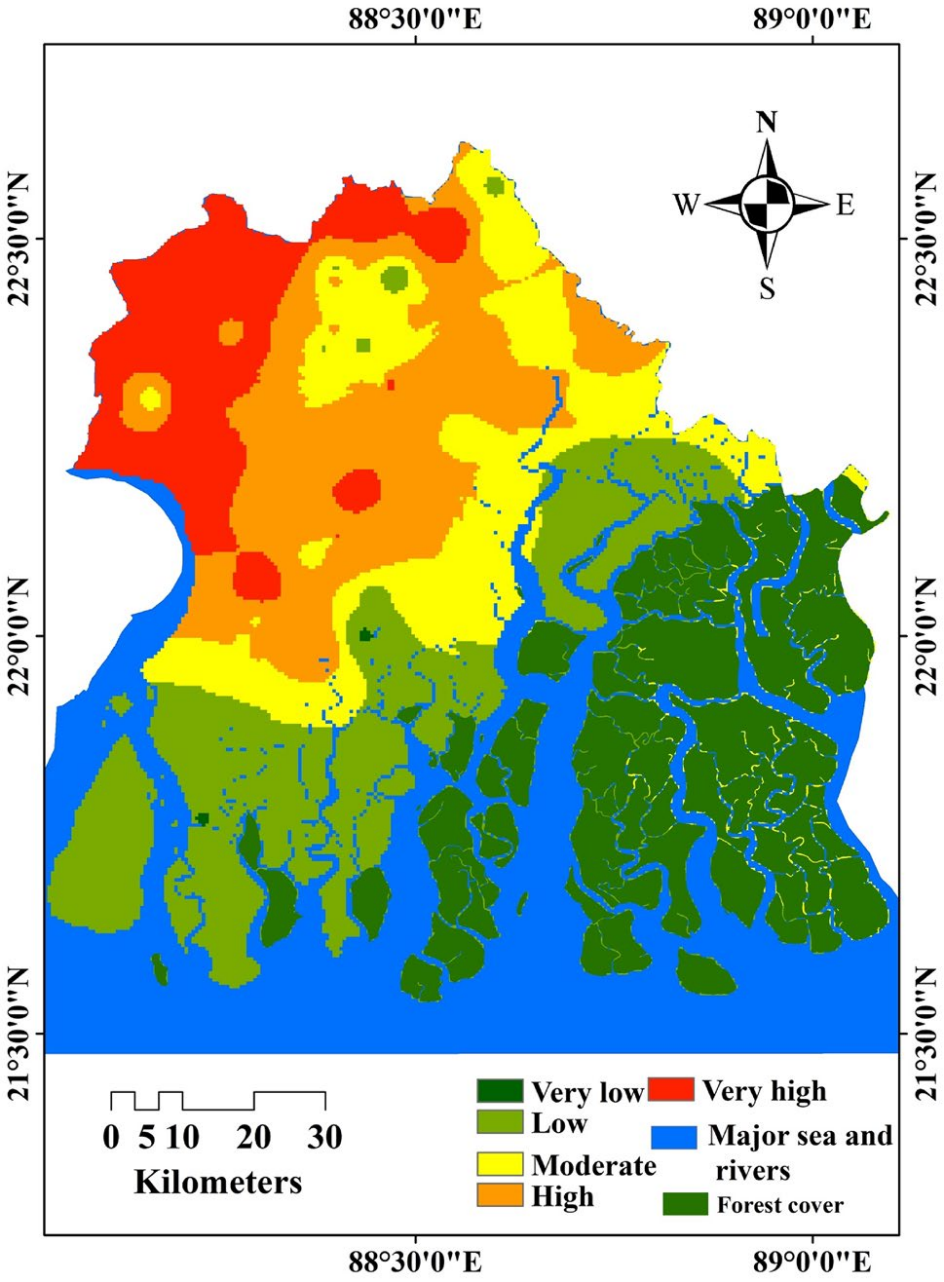
Population pressure (this map was generated using ArcGIS, version: 10.3.1, www.esri.com/arcgis).

In many countries, coastal tourism is increasing rapidly, so it negatively impacts coastal region's water, air and othernegatively affects coastal regions water, air and other environments^32^. In our study, we assessed the effect of population pressure on water quality. The population density of this district varies from one block to another. The average population density of this district is 819 sq/km., which is 214% more than the Indian population density. We classified five zones of stress on water quality like- very high, high, moderate, low, and very low. The North–western and northern part of this district is very high population pressure; southern islands of this district like Sagar Island, southern part of Namkhana etc., are less stress; the South–eastern and some north–eastern parts represent moderate stress, which is shown on (Fig. 5). Due to the density of this region, People suffer by pure drinking water scarcity. They depend only on shallow and deep tube wells for their daily potable water, and pond water is used for other activities like baths, toilet, etc. which is comparatively arsenic and fluoride contaminated. Due to this, contaminated water is the main source of drinking, so residents of this region suffer from several diseases like diarrhoea, kidney damage, and several diseases.

### Groundwater vulnerability and health risk analysis

**Figure 6:**
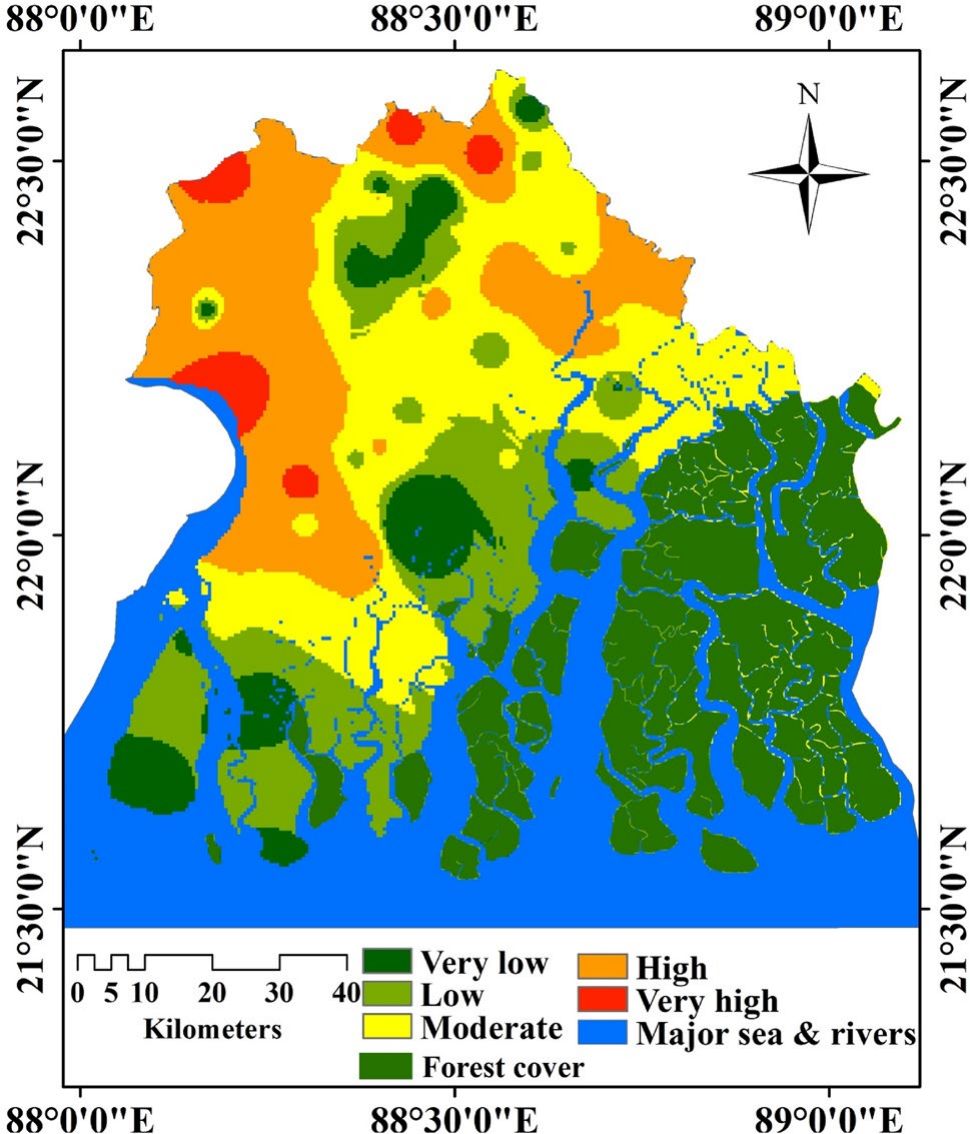
NO_3_ susceptibility (this map was generated using ArcGIS, version: 10.3.1, www.esri.com/arcgis).

In South 24 Parganas district, various patterns of health risk were observed. Some blocks represent high health risk, a few blocks representsrepresent high health risk, a few blocks represent high health risk, a few represent a high health risk, a few represent high health risk, and a few characterise low health risk. In this study area, five classes have been carried out like, very high, high, moderate, low and very low risk zones based on local conditions; because every location has distinctive locational settings, shown in Fig. 6. The derived result about groundwater vulnerability and corresponding health risk is fully controlled by regional geohydrological conditions as well as several environmental factors, including closeness to the ocean, geological settings, and aquifer depth, which significantly control this region's groundwater status. Maheshtola, Diamond Harbour II, Falta, Budge Budge, Western Bhangar very high health risks, and the north part of Kulpi represent very high health risks (Fig. 6); the southern part of Kulpi, some part of Patharpratima, Jaynagar I, II and north–eastern Canning II represents high human health hazard. A moderate human health hazard is observed in Baruipur, Magarhat II, major part of Gosaba and few part of Kakdwip. Major parts of this district like Sagar Island, the southern part of Namkhana, some parts of Basanti, and the southern portion of this study area fall under low human health hazard (Fig. 6). Result of Hazard quotient (HQ) for adult and children among four selected parameters is presented in Supplementary Table 1.

### Hydro-chemical properties

**Figure 7:**
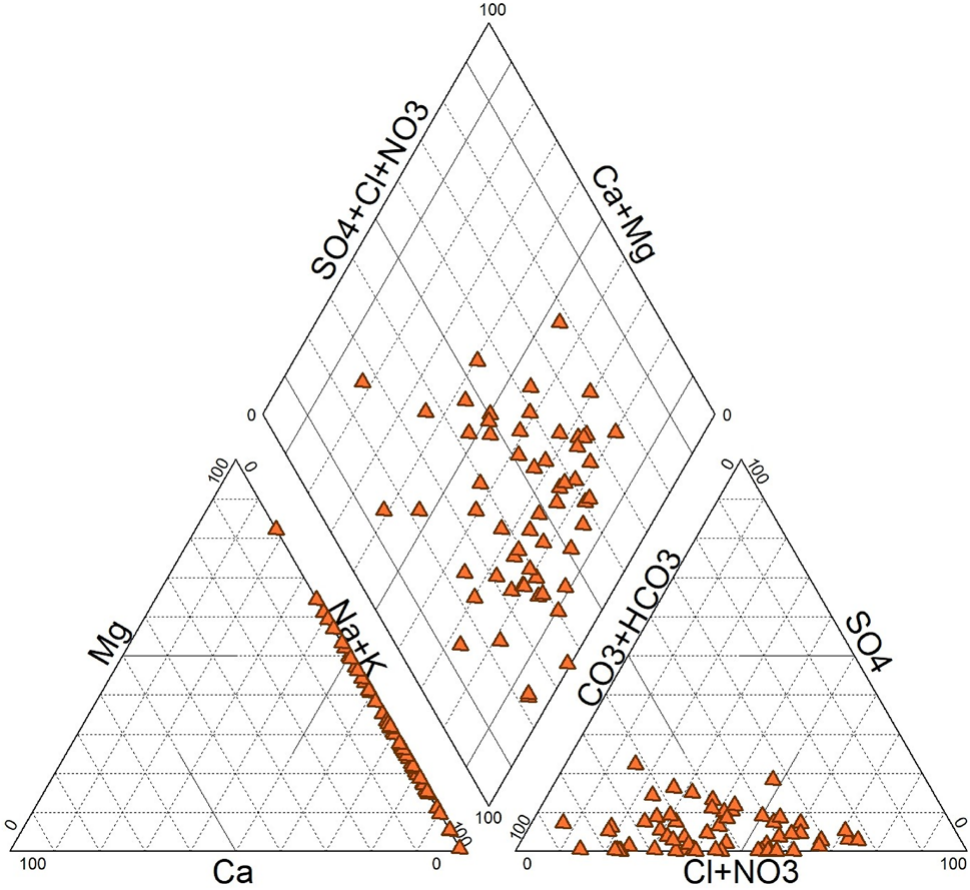
Piper diagram.

The Piper diagram can easily interpret the Chemistry of the water sample; sources of groundwater contamination can easily be predicted using the Piper diagram. The Piper diagram (Fig. 7) shows that the maximum samples fall under the alkaline type (Na++K+), which contains pH 8.5. Its characteristics are poor soil structure and low infiltration capacity. Sodium chloride and mixed types of samples are found in this study area. From the diagram (Fig. 7) we can predict that most wells have strong acids surpassing weak ones. Agriculture surface runoff is the main HCO_3_ source^33^; high exposure of Na^+^ increased in groundwater due to cation exchange capacity in clay. In groundwater, the highest concentration of alkaline organisms make water unfit for consumption.

### Model evaluation

**Figure 8:**
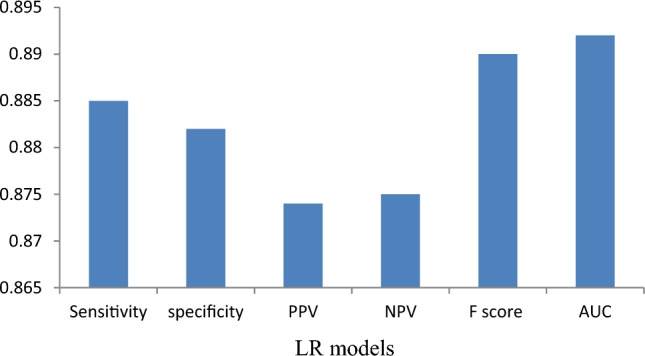
validating stage model evaluation through graphical presentation.

Appropriate validation procedures are essential to any scientific investigation; without them, the results obtained have no practical value. In this current research, six notable statistical validation methods have been employed, including specificity, sensitivity, positive predictive value (PPV), negative predictive value (NPV), F score and receiver operating characteristics curve (ROC)- area under curve (AUC) in validating the derived prediction measures with ground level; samples are used in two such as training and validating section. In these validation techniques, four distinctive parameters are applied, including true positive (TP), true negative (TN), false negative (FN), and false positive (FP) to estimate the validity of the result. These values from the validation procedure determine how accurate the adopted model are; greater values indicate better results from the model, and vice versa^34^. The validation results are shown in Table 3; among all the validating techniques AUC-ROC gives higher values 0.928 and 0.892 in training and validation section followed by specificity (training- 0.911, validation- 0.882), sensitivity (training- 0.915, validation- 0.885), PPV (training- 0.912, validation- 0.874), NPV (training- 0.91, validation- 0.875) and F score (training- 0.92, validation- 0.89). Therefore, the results state about the model accuracy; the adopted LR model is very much acceptable in this region according to geographical conditions; Fig. 8 shows the graphical representation of the performance of all adopted validating techniques.

**Table 3:** Values of model evaluation.

Models	Stage	Parameters					
		Sensitivity	Specificity	PPV	NPV	F-Score	AUC
	Training	0.915	0.911	0.912	0.91	0.92	0.928
	Validation	0.885	0.882	0.874	0.875	0.89	0.892

### Relative importance of causative factors

Mean Decrease Accuracy Method (MDA) is applied in this research work, and it is beneficial for ranking and choosing the factors of fourteen parameters related to nitrate concentration in groundwater. The very important factor is shown in Fig. 3. According to importance, these fourteen factors are ranked. Among all variables, pH is critical and ranked one, the value is 0.77, which highly controls the nitrate concentration in the coastal aquifer in South 24 Parganas; Cl occupies the second position- (value is 0.71) and other factors like—As, F^−^, EC and Mg^2+^ ranked third, fourth and fifth position with their value is 0.69, 0.69, 0.67 and 0.55, respectively. Other factors like—depth, temperature, and HCO_3_− are less influential factors for nitrate concentration in groundwater in this study, and their values are 0.32, 0.25 and 0.23, respectively. Moderately important factors are K^+^, SO_4_^2−^, and PO_4_^2−^ are moderate importance factors and their values are 0.53, 0.48 and 0.44, respectively. The overall study stated that all the selected causative factors are essential for nitrate concentration in the coastal groundwater aquifers of South 24 Parganas.

### Chemical analysis of coastal groundwater

**Figure 9:**
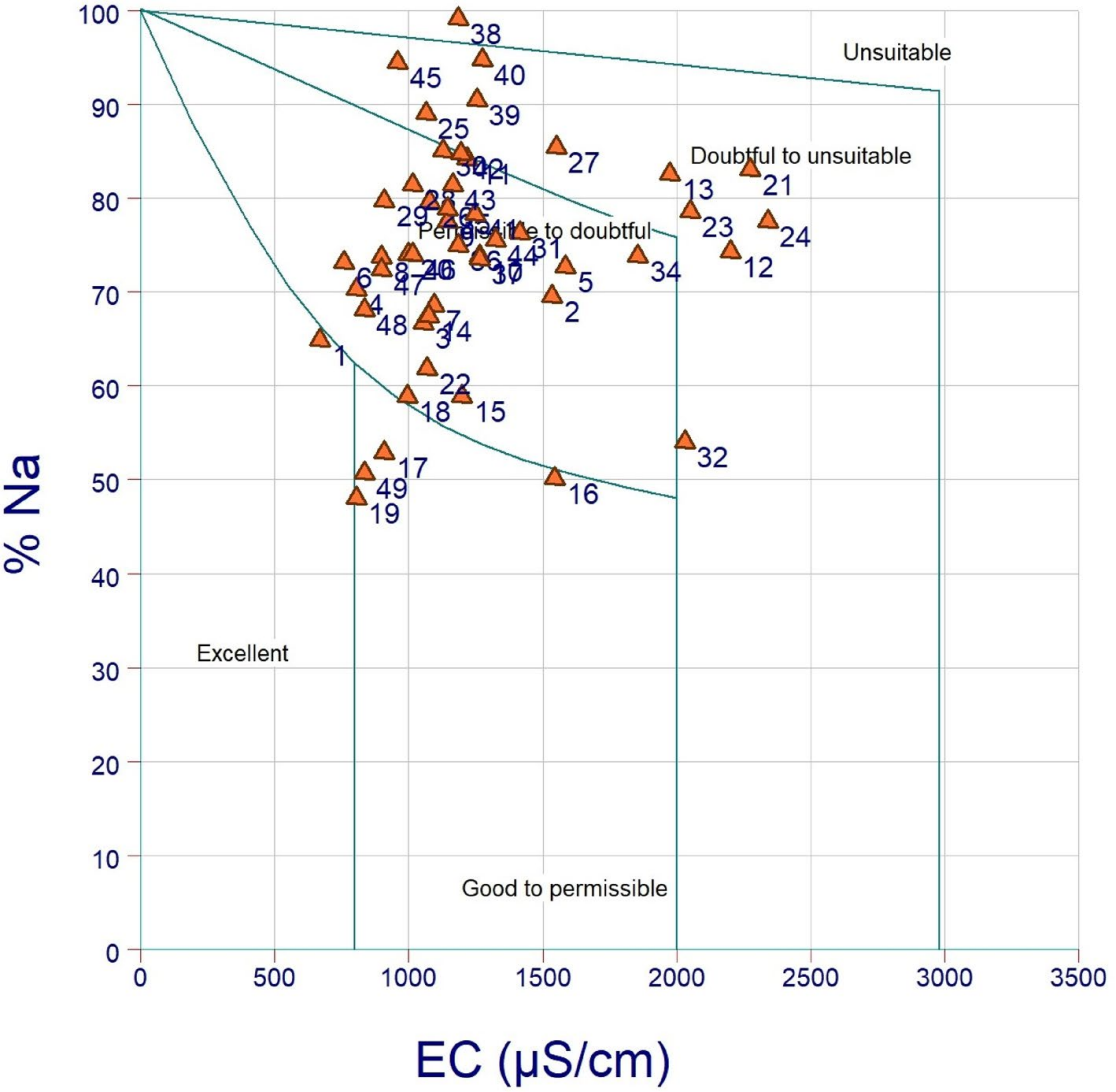
USSL diagram.

The USSL diagram is a plot between salinity hazard on the X axis and sodium hazard (SAR) on the Y axis which is proposed by “United State Salinity Laboratory (USSL)” for the classification of water which used for irrigation. This diagram (Fig. 9) classified water into 16 classes. For determine the salinity and sodium hazard 42 samples are selected. C3S1 represents medium salinity and low alkalinity which occupied 34.2%. C2S1 represents 17.02% total area, indicating moderate salinity and low alkalinity. C3S2 classes indicate high salinity and moderate alkalinity, representing 32% of tube wells32% of tube wells, and 32% of tube wells. Other important classes are C3S4 which indicates very high alkalinity and high salinity, which covered 12.76% total tube well. Only 2.12% tube well samples were covered by C4S4 represents very high alkalinity and salinity.

Wilcox diagram is an essential diagram for analysis the quality of groundwater. This diagram is categorized into five classes :- i. excellent to good ii. Good to permissible iii. Permissible to doubtful iv. Doubtful to unsuitable category and v. unsuitable category. FallThe highest percentage of data falls: The highest percentage falls under the acceptable to doubtful category (59.23%), then the doubtful to unsuitable category (27.27) and good to permissible category holds 9.09%; very few percentages occupied by excellent and unsuitable category (2.27%). It can be concluded that the highest number of samples are doubtful condition, so agriculture practices are threatened.

## Discussion

Identifying the hydro-chemical properties, eIdentifying the hydro-chemical properties, especially nitrate contamination, and its mitigation strategy in the coastal district in South 24 Pargana is an important work. In our research study, we identified the nitrate susceptibility map among all districts, and it depictss where the high, medium and low nitrate susceptibility occurred using the LR model. Different anthropogenic ies like industrial activity, agricultural activity, sewage etc. are highly correlated with groundwater nitrate concentration. Several researchers have shown that nitrate concentration is directly associated with different land-use patterns^35,36^. According to Kumazawa^37^ in agricultural activities use of nitrogen fertilizer create a great negative impact. Groundwater pollution and nitrate concentration are highly correlated with each other^38^.

Various studies still describe the hydro-chemical properties of groundwater and nitrate concentration susceptibility in the coastal district using other methodsstill describe the hydro-chemical properties of groundwater and nitrate concentration susceptibility in the coastal district using different methods and models like, LR. In our study a large proportion of area falls under the very high nitrate susceptibility zone. The total area is divided into five susceptibility zone including very high, high, moderate, low, and very low^39^ use RF and Genetic Algorithm (GA) for assessment of groundwater vulnerability. (Pal et al.^28^) used the RF and MDA method for determining the concentration of nitrate susceptibility prediction approach in coastal district. In our research study, we used fourteen nitrate conditioning factors. By using multi-collinearity analysis, we ranked them using MDA method. Among all variables, pH was essential and ranked one, value is 0.77 which is highly controlled the nitrate concentration in the coastal aquifer in South 24 Parganas followed by Cl^−^, value is 0.71. Other factors like—As, F^−^, EC and Mg^2+^ ranked third, fourth and fifth position and their value is 0.69, 0.69, 0.67 and 0.55, respectively. Other factors like depth, temperature, HCO_3_− are fewer effective factors for nitrate concentration in groundwater in this study, and their values are 0.32, 0.25 and 0.23 respectively. In our study the values of specificity, sensitivity, AUC and F score of training stage is greater (0.911, 0.915, 0.92 and 0.928) than validation stage. While, validation stages the values of sensitivity, specificity, F score and AUC are 0.885, 0.882, 0.89 and 0.892**,** which shows that the model is significantly applicable.

The nitrate concentration in South 24 Parganas district is very high, so different diseases like blue baby syndrome, fluorosis, diarrhoea and skin cancer are common in this area^40^. Many researchers have done research work about the coastal regions groundwater quality by using different methods like machine learning and GIS-based method^3,41,42^. To determine the health risk due to nitrate contamination we used acceptable field-based methods and techniques. (Pal et al.^28^) uses the same technique for assessing the nitrate susceptibility prediction approach in Indian coastal aquifers.

## Conclusions

Different parameters are used for determining the concentration of nitrate in coastal multi aquifers like—pH, Cl^−^, As, F^−^, EC, Mg^2+^, NO_3_−, K^+^, Temp., SO_4_^2−^, PO_4_^2−^, Na^+^, Salinity, Depth and HCO_3_−. Fifty-eight samples were used in this work; the highest relative important factor is pH (0.77) then Cl^−^ (0.71) and other variables like depth, temperature and HCO_3_− are less important than other factors. Concentration of nitrate in groundwater comes from several sources like, anthropogenic activities, agricultural activity, and sewage water etc. and its effects in coastal aquifer. In this research work we used data mining techniques like SPSS, Diagramme software, ArcGIS etc. to determine the nitrate concentration in coastal district, South 24 Parganas. The LR model is used to determine the nitrate concentration of this study area. In our study the values of specificity, sensitivity, AUC and F score of the training stage is greater (0.911, 0.915, 0.92 and 0.928) than validation stage. While validation stages the sensitivity, specificity, F score and AUC values are 0.885, 0.882, 0.89 and 0.892**,** which shows that the model is significantly applicable. In this region, some portions face nitrate concentration more than the rest of the portions. North–western, mid western and some part of northern portion is facing high nitrate concentrations. To determine the water quality and agricultural suitability for crop production, we used Piper’s diagram and USSL diagram. Different unscientific activities like, industry, agricultural practices and use of high chemical fertilizers also lead to high nitrate concentrations in this region. Another main problem in this region is saltwater intrusion in the agricultural field due to different naturally occurrings, cyclones, and floods. People of this region suffer by pure drinking water scarcity. They depend only on shallow and deep tube well for their daily potable water, which is comparatively arsenic and fluoride-contaminated. Due to this contaminated water is the primary source of drinking so residents of this region suffer by several diseases like diarrhoea, kidney damage, and several diseases. In this current research, we have several limitations. Firstly, we do not consider geology, soil type, land use, land cover pattern, and other hydrogeochemical parameters that may be responsible for nitrate concentration in an area. Still, here we have considered several nitrate conditioning factors that are incredibly accountable and mostly come from the abovementioned parameters. Secondly, only one model, LR, is used to determine the nitrate concentration of this coastal district. So, in the future, more advanced and scientific methods is applicable for predicting nitrate susceptibility. However, LR gives noteworthy ground truth prediction, which is quite similar to the actual condition of this region that also comes up in the result of all employed validating techniques. Therefore, this study is very similar to a ground scenario and accurately describes the existing alarming condition; thus, policymakers and stakeholder can take appropriate steps to reduce this lousy effect and create a healthy environment for the local people of this region.

### Supplementary Information

Supplementary Table 1.

## Data Availability

“The datasets used and/or analyzed during the current study are available by the corresponding author from the reasonable request”.
