# Supplementary material for: Source identification and potential health risks from elevated groundwater nitrate contamination in Sundarbans coastal aquifers, India
Source: Sci Rep. 2024 Feb 20;14:4153. doi: 10.1038/s41598-024-54646-0 (PMC10879081; doi:10.1038/s41598-024-54646-0)
Supplement: Supplementary file 1 — Supplementary Table 1. [file 41598_2024_54646_MOESM1_ESM.docx]

**Supplementary Table 1:** Result of Hazard quotient (HQ) for adult and children among four selected parameters

| Elements | Parameters | HQoral | |
| --- | --- | --- | --- |
|  |  | Adult | Children |
| As | Maximum | 4.19×10^-5^ | 3.61 |
|  | Minimum | 3.12×10^-2^ | 2.16 |
|  | Mean | 1.09×10^-1^ | 1.21 |
| F^-^ | Maximum | 5.32×10^-4^ | 3.21 |
|  | Minimum | 2.91×10^-4^ | 2.22 |
|  | Mean | 5.07 | 1.52 |
| Mg^2+^ | Maximum | 6.12 | 8.11 |
|  | Minimum | 1.92×10^-2^ | 3.14 |
|  | Mean | 1.16×10^-1^ | 1.34 |
| NO_3_^-^ | Maximum | 9.39 | 2.67 |
|  | Minimum | 7.68×10^-4^ | 2.20×10^-4^ |
|  | Mean | 8.12×10^-2^ | 4.18×10^-2^ |
